# Mechanically Robust and Room Temperature Self‐Healing Ionogel Based on Ionic Liquid Inhibited Reversible Reaction of Disulfide Bonds

**DOI:** 10.1002/advs.202207527

**Published:** 2023-05-01

**Authors:** Lei Yang, Lijie Sun, Hongfei Huang, Wenfan Zhu, Yihan Wang, Zekai Wu, Rasoul Esmaeely Neisiany, Shijia Gu, Zhengwei You

**Affiliations:** ^1^ State Key Laboratory for Modification of Chemical Fibers and Polymer Materials College of Materials Science and Engineering Institute of Functional Materials Donghua University, Research Base of Textile Materials for Flexible Electronics and Biomedical Applications (China Textile Engineering Society) Shanghai Engineering Research Center of Nano‐Biomaterials and Regenerative Medicine 2999 North Renmin Road Shanghai 201620 P. R. China; ^2^ Department of Materials and Polymer Engineering Faculty of Engineering Hakim Sabzevari University Sabzevar 9617976487 Iran

**Keywords:** disulfide bonds, ionic liquid, Ionogel, polyurethane, self‐healing

## Abstract

Although highly desired, it is difficult to develop mechanically robust and room temperature self‐healing ionic liquid‐based gels (ionogels), which are very promising for next‐generation stretchable electronic devices. Herein, it is discovered that the ionic liquid significantly reduces the reversible reaction rate of disulfide bonds without altering its thermodynamic equilibrium constant via small molecule model reaction and activation energy evolution of the dissociation of the dynamic network. This inhibitory effect would reduce the dissociated units in the dynamic polymeric network, beneficial for the strength of the ionogel. Furthermore, aromatic disulfide bonds with high reversibility are embedded in the polyurethane to endow the ionogel with superior room temperature self‐healing performance. Isocyanates with an asymmetric alicyclic structure are chosen to provide optimal exchange efficiencies for the embedded disulfide bonds relative to aromatic and linear aliphatic. Carbonyl‐rich poly(ethylene‐glycol‐adipate) diols are selected as soft segments to provide sufficient interaction sites for ionic liquids to endow the ionogel with high transparency, stretchability, and elasticity. Finally, a self‐healing ionogel with a tensile strength of 1.65 ± 0.08 MPa is successfully developed, which is significantly higher than all the reported transparent room temperature self‐healing ionogel and its application in a 3D printed stretchable numeric keyboard is exemplified.

## Graphical abstract

First the authors discovered that the ionic liquid significantly reduces the reversible reaction rate of disulfide bonds without altering its thermodynamic equilibrium constant. This inhibitory effect reduces the dissociated units in the dynamic polymeric network, critical for enhancing the strength of the ionogel. Accordingly, a room temperature self‐healing ionogel with a word record tensile strength of 1.65 ± 0.08 MPa is developed.

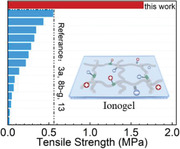

## 1 Introduction

Ionic liquid (IL)‐based gels (ionogels) refer to IL confined in three‐dimensional (3D) polymeric networks (We defined the IL content of ionogel should be greater than or equal to 40 wt% in this work).^[^ ^1^ ^]^ Ionogels hold great application prospects for wearable electronics,^[^ ^2^ ^]^ medical diagnosis,^[^ ^3^ ^]^ human–machine interfaces,^[^ ^4^ ^]^ and soft robotics^[^ ^5^ ^]^ due to their unique characteristics of outstanding stretchability, customizable electrical conductivity, and wide range of operating temperatures, as well as high thermal stabilities.^[^ ^1^, ^6^ ^]^ However, ionogels are susceptible to damage and lose their functions under complex deformations, leading to safety issues and waste of electronics.^[^ ^7^ ^]^

Room temperature self‐healing performance alludes to the ability of materials to heal themselves upon mechanical damage without the presence of extrinsic stimuli or additional substances for healing, which has drawn increasing attention in recent years.^[^ ^8^ ^]^ The durability and reliability of ionogels can be greatly improved by inducing the self‐healing property.^[^ ^9^ ^]^ A series of non‐covalent bonds (i.e., hydrogen bonds,^[^ ^10^ ^]^ ion bonds,^[^ ^11^ ^]^ and ion–dipole interactions,^[^ ^9a,b^ ^]^ etc.) have been introduced to construct self‐healing ionogels, but their low mechanical strength (<1 MPa) limits their further applications.

Here, we introduced aromatic disulfide bonds with higher reversibility (compared with aliphatic disulfide bonds) into the polyurethane network to endow the ionogel with superior room temperature self‐healing properties. The isophorone diisocyanate (IPDI) was selected as hard segment with a bulky asymmetric structure to prevent the crystallization of the polymer and increase the polymer chain mobility, thus further promoting the exchange of disulfide bonds. The soft segment adopted carbonyl‐rich poly(ethylene‐glycol‐adipate) diols, and the carbonyl groups provided sufficient interaction sites for the IL to endow the ionogel with high transparency, stretchability, and elasticity. Unexpectedly, we discovered that IL significantly reduced the reversible reaction rate of disulfide bonds without altering their thermodynamic equilibrium constant. This feature is critical for enhancing the mechanical strength of the resultant ionogel while keeping its self‐healing property. We also investigated its application using a 3D printed stretchable thin numeric keyboard.

## 2 Results and Discussion

**Figure 1:**
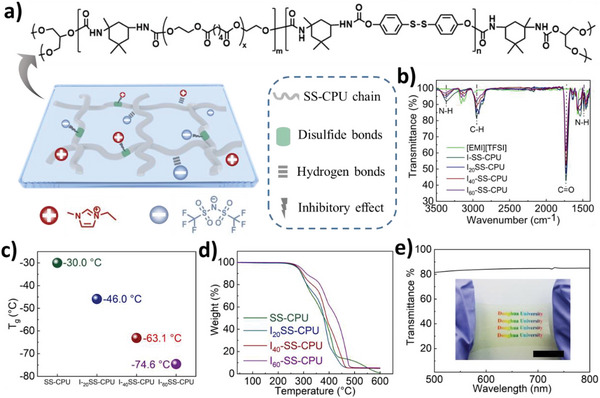
Design and characterization of the synthesized SS‐CPU and I‐SS‐CPUs. a) Molecular structures of [EMI][TFSI] and the SS‐CPU network and schematic illustration of their interaction. b) FTIR spectra of [EMI][TFSI], SS‐CPU, and the I‐SS‐CPUs (I_20_‐SS‐CPU, I_40_‐SS‐CPU, and I_60_‐SS‐CPU) containing 20, 40, and 60 wt% of IL. c) The determined *T*_g_ of SS‐CPU and I‐SS‐CPUs from DMA tests. d) TGA curves of the SS‐CPU and I‐SS‐CPU. e) Transmittance spectrum of I_40_‐SS‐CPU film (thickness: 1 mm). The average transmittance of I_40_‐SS‐CPU was over 84% in the wavenumber range of 500–800 nm. The inset photograph is the I_40_‐SS‐CPU film (Scale bar: 2 cm).

The design of disulfide bonds‐based crosslinking polyurethane ionogel (I‐SS‐CPU) is schematically illustrated in **Figure** **1**a. The I‐SS‐CPU includes extensive non‐covalent interactions (hydrogen bonding between IL 1‐ethyl‐3‐methylimidazolium bis (trifluoromethylsulfonyl) imide ([EMI][TFSI]) and polar group in polyurethane network) along with the inhibitory effect of [EMI][TFSI] on reversible reaction of disulfide bonds. The I‐SS‐CPU without IL is denoted as SS‐CPU. I_x_‐SS‐CPU refers to SS‐CPU with *x* wt% of the IL (concerning the weight of SS‐CPU). The H atoms of –NH in the I‐SS‐CPU and H atoms on imidazolium cation acted as hydrogen‐bond donors, while the strongly electronegative atoms including N, F, and O in the [TFSI] anion and C = O in I‐SS‐CPU functioned as hydrogen‐bond acceptors. Fourier‐transform infrared spectroscopy (FTIR) was utilized to examine the molecular interactions between [EMI][TFSI] and SS‐CPU networks. With the increase in the content of the IL, the C = O, and N–H of CPU chains stretching vibrations moved from 1728 and 3363 cm^−1^ to 1730 and 3386 cm^−1^ in the I‐SS‐CPU (Figure 1b), respectively. In addition, the peaks located at 1346, 1177, 1132, and 1049 cm^−1^ corresponded to the O = S = O asymmetric, CF_3_, O = S = O symmetric, and S–N–S stretches vibration of the [EMI][TFSI] in I‐SS‐CPUs shifted to 1351, 1183, 1134, and 1054 cm^−1^, respectively (Figure S1, Supporting Information). These results revealed that the hydrogen bonds between the carbonyl and carbamate groups of the CPU chains were replaced by an interaction between the polymeric chains and the IL. Moreover, the strong interaction between the IL and the PU chain likely facilitated the dispersion of [EMI][TFSI] in the PU network and prevented its leaking from ionogels. As shown by dynamic mechanical analysis (DMA) (Figure S2, Supporting Information), the glass transition temperature (*T*_g_) of the I‐SS‐CPU reduced with increasing [EMI][TFSI] content (Figure 1c). I_60_‐SS‐CPU had a much lower *T*_g_ (−74.6 °C) than SS‐CPU (−30.0 °C), indicating the lubricating effect of [EMI][TFSI] on polymer network. As shown in Figure 1d, SS‐CPU and I‐SS‐CPU exhibited excellent thermal stability. The thermal decomposition temperatures of SS‐CPU, I_20_‐SS‐CPU, I_40_‐SS‐CPU, and I_60_‐SS‐CPU were determined to be 275.9, 276.6, 280.3, and 289.1 °C, respectively. In addition, we observed that the mass of the I_40_‐SS‐CPU remained almost constant over a period of 28 days (Table S1, Supporting Information). Meanwhile, the FTIR spectra were essential unchanged including the characteristic absorption bands of the PU chain and [EMI][TFSI]. These results indicated the remarkable stability of I_40_‐SS‐CPU. Furthermore, the high miscibility of the [EMI][TFSI] with polymer networks led to the I_40_‐SS‐CPU with high transparency. I_40_‐SS‐CPU had an average transmittance of over 84% for a 1‐mm‐thick film under visible‐light wavelengths of 500–800 nm (Figure 1e). In addition, I_40_‐SS‐CPU did not dissolve in tetrahydrofuran, dimethylacetamide, and acetone, indicating its crosslinking structure (Figure S4, Supporting Information).

**Figure 2:**
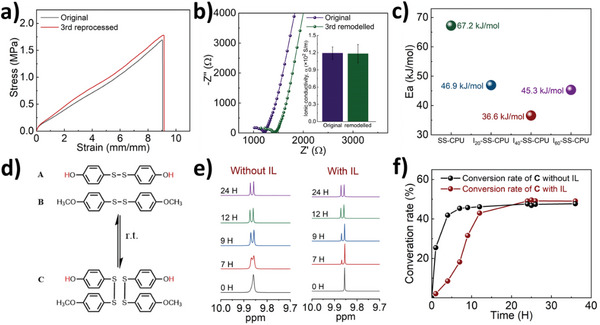
Dynamic properties of I‐SS‐CPU based on IL inhibited reversible reaction of disulfide bonds. a) Stress–strain curves of the original and 3rd reprocessed I_40_‐SS‐CPU showed full recovery of the mechanical property. b) Electrochemical impedance spectra of original and 3rd reprocessed I_40_‐SS‐CPU (inset: the conductivity of original and reconfiguration I_40_‐SS‐CPU). c) The activation energies of the SS‐CPU and I‐SS‐CPU. d) Scheme of dynamic exchange reaction in disulfide bonds bearing small‐molecule model. e) ^1^H NMR spectra of mixture compound **A** and **B** with and without [EMI][TFSI] for different reaction times at 25 °C. f) Conversion rate of compound **C** with and without [EMI][TFSI] (conversion ratio = [**C**]_t_/([**A**]_0_ + [**B**]_0_), [**C**]_t_ = concentration of compound **C** at time *t*, [**A**]_0_ = original concentration of compound **A**, [**B**]_0_ = original concentration of compound **B**.

Owing to their wide use and limited‐service life, electronic pollution has become a rapidly growing global problem. Therefore, it is a high desire to develop recyclable electronics to decrease the environmental and economic burdens of such waste.^[^ ^12^ ^]^ Given the presence of highly dynamic chemical structures and the mobility of the polymer network, the I_40_‐SS‐CPU exhibited excellent recyclability. This property was evaluated by remolding chopped pieces of I_40_‐SS‐CPU (**Figure** **2**a and Figure S5, Supporting Information). In addition, the stress‐strain curves of I_40_‐SS‐CPU before and after 3 times reprocessing were similar. Furthermore, we investigated the structural and electronic properties of I_40_‐SS‐CPU before and after reprocessing to verify the reconfiguration property. FTIR spectra showed that the reprocessed I_40_‐SS‐CPU maintained its original chemical structures (Figure S6, Supporting Information). Moreover, the ionic conductivity of reprocessed I_40_‐SS‐CPU (1.19 ± 0.11 × 10^−2^ S m^−1^) was negligible change in comparison with the original (1.18 ± 0.16 × 10^−2^ S m^−1^), which showed excellent reconfigurability of I_40_‐SS‐CPU (Figure 2b). Reconfigurability is closely related to the activation energy of the dissociation of the dynamic covalent network.^[^ ^13^ ^]^ To further investigate the influence of IL on the reconfiguration characteristics of polymers, the rheological properties of the I_40_‐SS‐CPU were measured by stress‐relaxation tests at different temperatures (Figure S7, Supporting Information). For the stress‐relaxation examination, the relaxation modulus was measured as a function of time, while a torsional strain of 5% was applied. With the increase in temperature, the relaxation times of SS‐CPU and I‐SS‐CPU decreased. According to the Maxwell model for viscoelastic fluids, the relaxation times (τ*) were determined at 37% (*G*/*G*_0_ = 1/*e* ≈ 37%) of the normalized relaxation modulus. The temperature dependence of the relaxation time could be illustrated by the Arrhenius equation: *τ* (*T*) = *τ*_0_ exp(*E*_a_/*RT*), where *τ* refers to the characteristic relaxation time, *τ*_0_ is attributed to the pre‐exponential factor, and *E*_a_ corresponds to the stress relaxation activation energy. The *E*_a_ of SS‐CPU, I_20_‐SS‐CPU, I_40_‐SS‐CPU, and I_60_‐SS‐CPU were determined to be 67.2, 46.9, 36.6, and 45.3 kJ mol^−1^, respectively (Figure S8, Supporting Information and Figure 2c). The *E*_a_ significantly decreased before the content of the IL in ionogels increased to 40%. These results confirmed that the IL had a significant lubricating effect on polymer networks. Unexpectedly, the *E*_a_ of the I_60_‐SS‐CPU was higher than that acquired for the I_40_‐SS‐CPU. It might be attributed to the inhibitory effect of IL on reversible reaction of disulfide bonds. To further reveal the mechanism of disulfide bond exchange reaction with and without IL, electron paramagnetic resonance (EPR) measurements were used to detect the sulfur radical in the SS‐CPU and I‐SS‐CPUs (Figure S9, Supporting Information). The *g*‐values in the ESR derivatives around 2.119 likely corresponded to sulfur radicals. Additionally, the peak intensity was positively correlated with the content of free radicals, which was consistent with the change in activation energy. It may be attributed to the inhibitory effect of IL on the reversible reaction of disulfide bonds.

A small molecules model was used to demonstrate the inhibitory effect of IL on the reversible reaction of disulfide bonds. As shown in Figure 2d, compound **A**‐bis(4‐methoxyphenyl) disulfide and **B**‐bis(4‐hydroxyphenyl) disulfide, both containing disulfide bonds, were mixed. The ^1^H nuclear magnetic resonance (NMR) spectrum of compound **A** in the mixture showed proton signals of the hydroxyl at 9.86 ppm (Figure 2e). With time, a new proton signal at 9.85 ppm was observed, which can be attributed to the product 4‐((4‐methoxyphenyl) disulfanyl) phenol (compound **C**), indicating the occurrence of an exchange reaction. The reaction took ≈9 h to reach equilibrium at 25 °C (Figure 2f). Next, [EMI][TFSI] was added to the aforementioned exchange reaction. It took ≈20 h to reach equilibrium, which is much slower than the non‐incorporated IL sample. Specifically, the dissociation kinetics of the disulfide bond without and with ionic liquid in this small molecular reaction were 0.102 and 0.066 h^−1^, respectively (Figure S10, Supporting Information). It demonstrated the inhibitory effect of IL on the reaction rate of reversible disulfide bonds. However, the reversible reaction endpoints (with and without IL) of disulfide bonds were almost similar. To further verified the effect of ionic liquids on the thermodynamics of disulfide bond exchange reaction, in situ variable‐temperature ^1^H NMR spectra of small molecular model reaction were used to calculate equilibrium constants (Figures S11 and S12, Supporting Information). At 20 °C, the chemical equilibrium constants of the exchange reaction of the small molecules model with and without ionic IL were 0.9981 and 1.0187, respectively. Additionally, the trend of the chemical equilibrium constant of the exchange reaction of small molecules model with IL was similar to without IL with the increasing temperature. These results demonstrated that the IL did not alter either the thermodynamic equilibrium constant of dynamic disulfide bonds.

**Figure 3:**
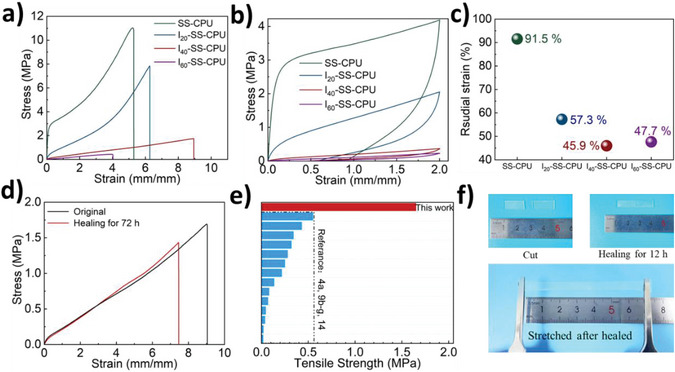
Mechanical properties and self‐healing performance of SS‐CPU and I‐SS‐CPUs. a) Representative stress–strain curves of the SS‐CPU and I‐SS‐CPUs. b) Tensile loading–unloading curves of SS‐CPU and I‐SS‐CPUs at 200%. c) Residual strains of the SS‐CPU and I‐SS‐CPUs after tensile loading‐unloading at 200%. d) Typical tensile stress–strain plots of original and room temperature healed (after 72 h of the healing process)I_40_‐SS‐CPU. e) Ashby plot of tensile strength of I_40_‐SS‐CPU and other room temperature self‐healing ionogels reported in the literature.^[^ ^4^, ^9^, ^14^ ^]^ f) A photograph of cut and healed I_40_‐SS‐CPU film with the ability to stretch to twice its original length, after healing for 12 h could be stretched to twice its original length.

Next, the mechanical properties and self‐healing performance of SS‐CPU and I‐SS‐CPU were investigated. The hydrogen bonds in the original polymer network were replaced by hydrogen bonds between IL and the polymer network. It effectively enhanced the mobility of the PU chains and tuned the mechanical properties of SS‐CPU. Uniaxial and loading‐unloading tensile tests were conducted to evaluate the mechanical properties of the I‐SS‐CPU with different IL contents. SS‐CPU showed a tensile strength and a strain at fracture of 11.46 ± 1.23 MPa and 541 ± 34%, respectively (**Figure** **3**a). However, a bulky hysteresis loop was detected during the tensile loading‐unloading test, indicating the poor elasticity of the I‐SS‐CPU (Figure 3b). This large hysteresis can be ascribed to the hydrogen bonds in PU chains that hampered the resilience of the SS‐CPU after stretching. After the introduction of the IL, the hydrogen bonds between the PU chains were largely replaced by the hydrogen bonds between [EMI][TFSI] and the PU chains. Consequently, the hysteresis loop of the I‐SS‐CPU was significantly reduced. At the same time, the I‐SS‐CPU showed higher stretchability and lower Young's modulus than those acquired for the SS‐CPU. For example, I_40_‐SS‐CPU had a tensile strength, maximum extensibility, and Young's modulus of 1.65 ± 0.08 MPa, 900%, and 283 ± 15 kPa, respectively (Figure 3a). Furthermore, the elasticities of the I‐SS‐CPU were quantitatively characterized in terms of the residual strain after the tensile loading–unloading at a strain of 200% (Figure 3c). Compared to the SS‐CPU (with a residual strain of 91.5%), the I‐SS‐CPU exhibited significantly better elasticity with much smaller residual strains. When the amount of loaded IL further increased to 60%, the residual strain of I_60_‐SS‐CPU (47.7%) was slightly increased than that of I_40_‐SS‐CPU (45.9%), because the excess IL increased the viscosity, instead of the elasticity, of the material.

The self‐healing property of I_40_‐SS‐CPU was also evaluated in detail. For its bulky self‐healing (Figure 3d), the rectangle samples of I_40_‐SS‐CPU were firstly cut into two pieces, then connected at room‐temperature. After 72 h, its tensile strength recovered to 91.6% (1.51 ± 0.14 MPa) of the original stress (1.65 ± 0.08 MPa), which is significantly more than the original tensile strength of all previously reported self‐healing unadulterated ionogels^[^ ^4^, ^9^, ^14^ ^]^ and the strain recovered to 82.7% (740 ± 10%) of the original (890 ± 20%) (Figure 3e). Additive materials such as small molecules can also be added to ionogels to enhance tensile strength. This approach mostly led to reducing their transparency and limiting their applications in display devices and electroluminescent devices, and so on. I_40_‐SS‐CPU with such excellent mechanical and self‐healing properties may attribute to the inhibitory effect of [EMI][TFSI] on the reversible reaction of disulfide bonds. IL significantly reduced the reversible reaction rate of disulfide bonds. Thus, the dissociated units in the dynamic polymeric network were reduced, beneficial for the strength of the ionogel. However, IL unchanged the thermodynamic equilibrium constant of dynamic disulfide bonds. Therefore, the IL did not reduce the healing rate (the percentage of the recovery of mechanical properties, mainly including the tensile strength, elongation, and toughness, after healing) of the I_40_‐SS‐CPU. The relatively short‐term self‐healing ability of I_40_‐SS‐CPU was evaluated by healing after 12 h (Figure 3f).

**Figure 4:**
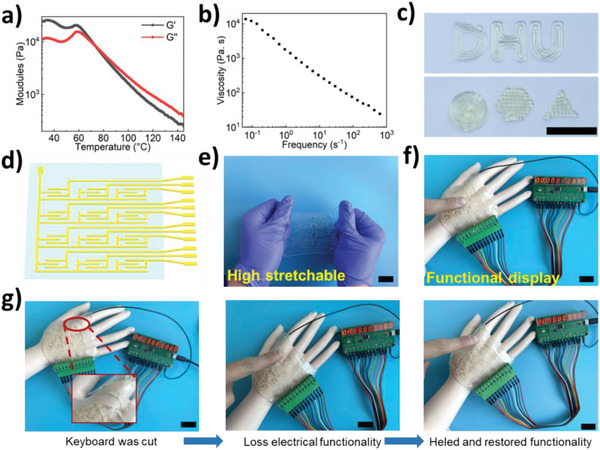
The electrical properties and 3D printability of I_40_‐SS‐CPU. a) Storage modulus, loss modulus, and b) complex viscosity of the I_40_‐SS‐CPU versus temperature. c) Photographs of 3D printed alphabetic letters, circles, hexagons, and triangles. d) Schematic of the stretchable keyboard. e) Demonstration of the stretchability of the flexible keyboard. f) Photograph of numeric keyboard with a functional display. g) The flexible keyboard was cut off and lose electrical functionality. Then, the stretchable keyboard was healed and restored the electrical function after 1 h (Scale bar: 2 cm).

As presented in **Figure** **4**a, the rheological results, including storage modulus (*G*′) and loss modulus (*G*″), demonstrated that the transition temperature from an elastomeric network state (*G*′ > *G*″) to a viscoelastic liquid state (*G*′ < *G*″) was 74 °C. The shear‐thinning property was also observed through the frequency scanning tests. With the increase in frequency at 120 °C, the viscosity of the polymer gradually decreased (Figure 4b). These demonstrated that the I_40_‐SS‐CPU has excellent 3D printability at 120 °C. Subsequently, the 3D‐printed letters “D,” “H,” and “U,” and the geometrical shapes of circle, hexagon, and triangle further confirmed the great printability of I_40_‐SS‐CPU (Figure 4c). Furthermore, a stretchable thin numeric keyboard was designed and 3D printed (VHB was used as the printing substrate) for wearable user input interfaces, which are highly demanded in next‐generation stretchable electronics (Figure 4d,e). The function of the keyboard was realized through circuit conduction. Upon the key being pressed, the circuit was connected, and the associated number would then be displayed and recorded. As shown in Figure 4f, the numeric keyboard was easily attached to the hand. Furthermore, to evaluate the self‐healing property of the numeric keyboard, a fracture was created; therefore, the function of the numeric keypad suddenly failed (Figure 4g). After healing for 1 h, the electrical property of the numeric keypad was restored. Overall, I_40_‐SS‐CPU holds great promise in customizable self‐healing stretchable electronic devices.

## 3 Conclusions

In summary, we successfully developed a room temperature transparent self‐healing ionogel with a world‐record tensile strength, which shows great potential in emerging stretchable electronic devices. Distinguishing from most existing ionogels, which are noncovalently crosslinked, the dynamic disulfide bonds‐based covalent network provides superior mechanical properties while endowing it with excellent self‐healing ability. Especially, we discovered that IL reduced the exchange reaction rate of reversible disulfide bonds, which may contribute to the high tensile strength of ionogel. Existing studies on dynamic polymers focus on enhancing the reversibility of dynamic bonds, while this newly discovered inhibitory effect will provide a new paradigm to construct materials with unusual properties. Generally, IL has been widely used as a catalyst to enhance the chemical reaction rate. This is the first report that IL reduces the chemical reaction rate. It will initiate a new direction for IL and may lead to a new field of chemistry and materials.

## 4 Experimental Section

### Material

Isophorone diisocyanate (IPDI), bis(4‐methoxyphenyl) disulfide, and dibutyltin dilaurate (DBTDL, 95%) were all purchased from Aladdin. Furthermore, 1‐ethyl‐3‐methylimidazolium bis(trifluoromethylsulfonyl) imide ([EMI][TFSI]), ≥ 99%) was obtained from the Lanzhou Institute of Chemical Physics, CAS. Bis(4‐hydroxyphenyl) disulfide (98%) was supplied by TCI (Japan). Glycerol was purchased from Sigma‐Aldrich. Poly(ethylene‐glycol‐adipate) diols (PEGAD, *M*_w_ ≈ 1000) were purchased from Jining Baichuan Chemical Co. Ltd. Deuterated solvents were purchased from Cambridge Isotope Laboratories, Inc. Other solvents were obtained from Sinopharm Chemical Reagent. All the reagents were employed as received with no further purification except otherwise mentioned.

### Preparation of the SS‐CPU and I‐SS‐CPU

PEGAD (12.0 g, 12 mmol) was filled into a glass reactor and dried at 110 °C for 2 h under a vacuum. Then cooling to 70 °C, IPDI (9.5 g, 28.5 mmol) and DBTDL (43 mg) were dissolved in DMAC (6 mL), then added to the mixture to react for 2 h, while the mixture was magnetically stirred during the reaction. The mixture was allowed to be cooled to 25 °C, then bis(4‐hydroxyphenyl) disulfide (3.0 g,12 mmol), glycerol (276 mg, 3 mmol), and DMAC(15 mL) were decanted into the mixture and heated to 40 °C for 17 h. Next, DMAC(15 mL) and equivalent required [EMI][TFSI] were gradually added to the reactor for 0.5 h. Subsequently, the obtained mixture was poured into a polytetrafluoroethylene mold and gradually heated to 120 °C for 32 h. The residual solvent was eliminated using a vacuum oven at 60 °C for 3 days to produce SS‐CPU and I‐SS‐CPU.

### Determination of Equilibrium Constants

Compounds **A**, **B**, and [EMI][TFSI] were dissolved in DMSO‐_d6_ (0.6 mL) for NMR measurement (The control group did not add [EMI][TFSI]). ^1^H NMR spectra were collected at variable‐temperatures. The concentration of each compound was calculated by the integral ratios of ^1^H NMR signals and the initial concentrations of **A** and **B**. The equilibrium constants were calculated following equation:

(1) $$K_{\mathrm{eq}}=\frac{{\left[C\right]}_{\mathrm{eq}}^{2}}{{\left[A\right]}_{\mathrm{eq}}{\left[B\right]}_{\mathrm{eq}}}\;$$

### 4.1 Printing of Electronics

The 3D printing was performed using a commercially available pressure‐controlled direct ink 3D printing system (BS4.2, GESIM). The mechanical‐driven extrusion process used micronozzles with a 500 µm inner diameter. All printing paths were controlled by the operational software (GesimRobotics). All the 3D models were designed by 3D Builder and GesimRobotics software.

### Characterization and Measurement

^1^H NMR spectra of the samples were measured using a Bruker AVANCE 600 NMR spectrometer. The electron paramagnetic resonance (EPR) measurements were performed on a Bruker EMXplus. The attenuated total reflectance FTIR (ATR‐FTIR) spectra were also recorded employing a Thermo Scientific Nicolet 8700 spectrometer. The mechanical properties of the samples were assessed by an MTS E42 tensile machine equipped with a 100 N load cell. The uniaxial tensile tests were carried out while the crosshead was adjusted at 50 mm min^−1^. The electrochemical properties of the samples were assessed using a CHI670E electrochemical analyzer. To determine the optical transmittance of the samples, a Jasco V‐630 UV–visible spectrophotometer was employed. The thermal stability of the prepared ionogels was evaluated using a series of TGA tests using a TG 209 F1 thermogravimetric analyzer (NETZSCH, Germany) in the temperature range of 40–600 °C with a heating rate of 10 °C min^−1^ under a nitrogen atmosphere. Stress‐relaxation tests were then performed in a TA Instruments ARES‐G2 rheometer with a 25 mm parallel plate geometry for SS‐CPU, and I‐SS‐CPU specimens with thicknesses of 1 mm by applying 5% strain at a constant gap. Flow analyses were also performed employing a TA Instruments ARES‐G2 rheometer with a strain of 0.5% and a heating rate of 3 °C min^−1^. The *T*_g_ of the prepared ionogels was determined to employ a dynamic mechanical analyzer (DMA, Thermal Analysis DMA‐Q800). For the DMA tests, the rectangular specimens were evaluated at heating ramps of 5 °C min^−1^, while the frequency and stain of the tests were set at 1 Hz and 0.1%, respectively.

## Conflict of Interest

The authors declare no conflict of interest.

## Supporting information

Supporting Information

## Data Availability

The data that support the findings of this study are available from the corresponding author upon reasonable request.

## References

[1] C. Keplinger , J.‐Y. Sun , C. C. Foo , P. Rothemund , G. M. Whitesides , Z. Suo , Science 2013, 341, 984. 23990555 10.1126/science.1240228

[2] L. Sun , H. Huang , Q. Guan , L. Yang , L. Zhang , B. Hu , R. E. Neisiany , Z. You , M. Zhu , CCS Chem. 2022, 10.31635/ccschem.022.202202037.

[3] X. Wang , Z. Liu , T. Zhang , Small 2017, 13, 1602790. 10.1002/smll.201602790 28306196

[4] a) J. Zhang , E. Liu , S. Hao , X. Yang , T. Li , C. Lou , M. Run , H. Song , Chem. Eng. J. 2022, 431, 133949;

[5] a) Y. Gu , E. A. Alt , H. Wang , X. Li , A. P. Willard , J. A. Johnson , Nature 2018, 560, 65; 30022167 10.1038/s41586-018-0339-0

[6] a) S. J. Zhang , N. Sun , X. Z. He , X. M. Lu , X. P. Zhang , J. Phys. Chem. Ref. Data 2006, 35, 1475;

[7] a) W. Zhang , H. Jiang , Z. Chang , W. Wu , G. Wu , R. Wu , J. Li , J. Mater. Sci. 2020, 55, 13543;

[8] a) S. Zheng , W. Li , Y. Ren , Z. Liu , X. Zou , Y. Hu , J. Guo , Z. Sun , F. Yan , Adv. Mater. 2022, 34, 2106570; 10.1002/adma.202106570 34751468

[9] a) Y. Cao , T. G. Morrissey , E. Acome , S. I. Allec , B. M. Wong , C. Keplinger , C. Wang , Adv. Mater. 2017, 29, 1605099; 10.1002/adma.201605099 28009480

[10] a) M. Zhu , S. He , Y. Dai , J. Han , L. Gan , J. Liu , M. Long , ACS Sustainable Chem. Eng. 2018, 6, 17087;

[11] P. Shi , Y. Wang , W. W. Tjiu , C. Zhang , T. Liu , ACS Appl. Mater. Interfaces 2021, 13, 49358. 34632775 10.1021/acsami.1c16081

[12] B. H. Robinson , Sci. Total Environ. 2009, 408, 183. 19846207 10.1016/j.scitotenv.2009.09.044

[13] a) C. He , S. Shi , D. Wang , B. A. Helms , T. P. Russell , J. Am. Chem. Soc. 2019, 141, 13753; 31433176 10.1021/jacs.9b06668

[14] a) K. Parida , V. Kumar , W. Jiangxin , V. Bhavanasi , R. Bendi , P. S. Lee , Adv. Mater. 2017, 29, 1702181; 10.1002/adma.201702181 28744921

