# Supplementary material for: Mechanically Robust and Room Temperature Self‐Healing Ionogel Based on Ionic Liquid Inhibited Reversible Reaction of Disulfide Bonds
Source: Adv Sci (Weinh). 2023 May 1;10(20):2207527. doi: 10.1002/advs.202207527 (PMC10369268; doi:10.1002/advs.202207527)
Supplement: Supplementary file 1 — Supporting Information [file ADVS-10-2207527-s001.pdf]

ADVANCED SCIENCE
Open Access

Supporting Information

for *Adv. Sci.*, DOI 10.1002/advs.202207527

Mechanically Robust and Room Temperature Self-Healing Ionogel Based on Ionic Liquid Inhibited Reversible Reaction of Disulfide Bonds

*Lei Yang, Lijie Sun, Hongfei Huang, Wenfan Zhu, Yihan Wang, Zekai Wu, Rasoul Esmaeely Neisiany, Shijia Gu and Zhengwei You**

Supporting information for

Mechanically robust and room temperature self-healing ionogel based on ionic liquid inhibited reversible reaction of disulfide bonds

Lei Yang, [1] *Lijie Sun,* [1] *Hongfei Huang,* [1] *Wenfan Zhu,* [1] *Yihan Wang,* [1] *Zekai Wu,* [1] *Rasoul Esmaeely Neisiany*[2], *Shijia Gu*[1] *and Zhengwei You*[1]*

[1]State Key Laboratory for Modification of Chemical Fibers and Polymer Materials, College of Materials Science and Engineering, Institute of Functional Materials, Donghua University, Research Base of Textile Materials for Flexible Electronics and Biomedical Applications (China Textile Engineering Society), Shanghai Engineering Research Center of Nano-Biomaterials and Regenerative Medicine, 2999 North Renmin Road, Shanghai 201620, P. R. China.

[2]Department of Materials and Polymer Engineering, Faculty of Engineering, Hakim Sabzevari University, Sabzevar 9617976487, Iran.

*Corresponding Author. E-mail: zyou@dhu.edu.cn (Z. Y.)

Time (Day)	0	7	14	21	28
Mass (g)	0.9381	0.9384	0.9395	0.9388	0.9390

Table S1. The mass change of I_{40}-SS-CPU as a function of time.

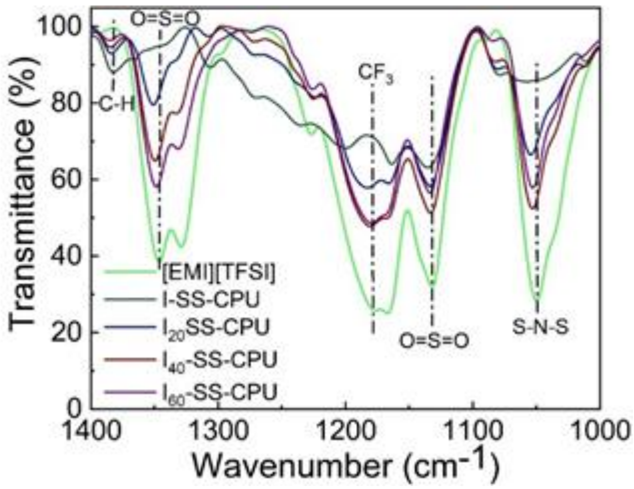

Figure S1. The fourier transform infrared spectrometer (FTIR) spectra of [EMI][TFSI], SS-CPU, and I-SS-CPUs.

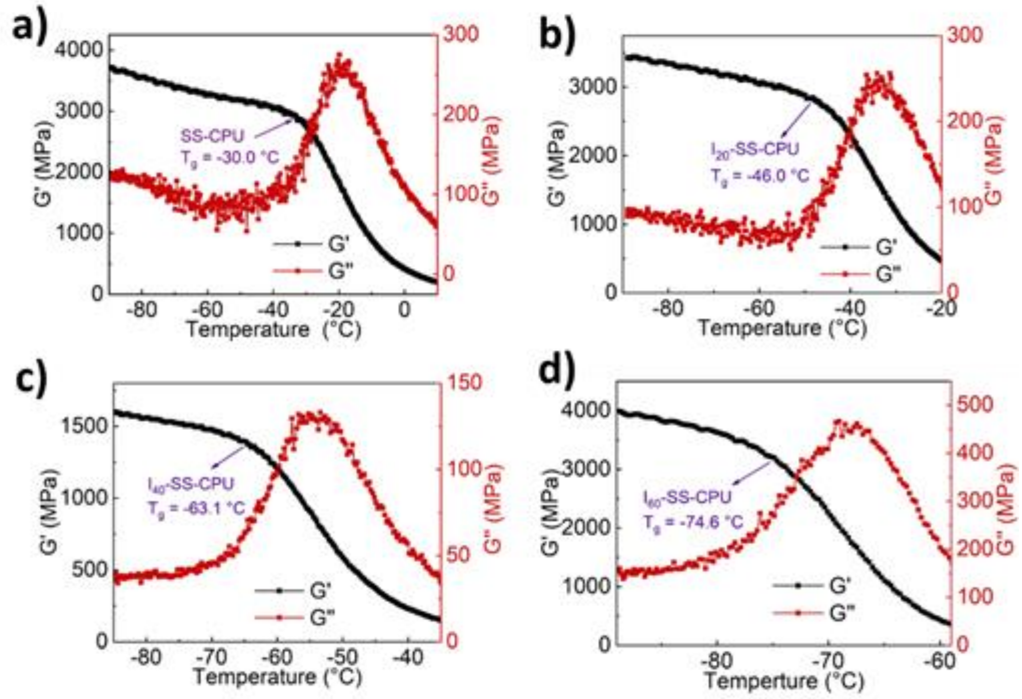

Figure S2. The dynamic mechanical analysis curves of a) SS-CPU, b) I_{20}-SS-CPU, c) I_{40}-SS-CPU, and d) I_{60}-SS-CPU.

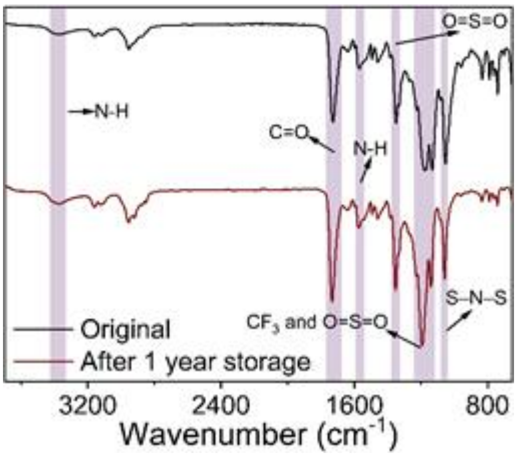

Figure S3. FTIR spectra of I_{40}-SS-CPU in 0 and 491 days.

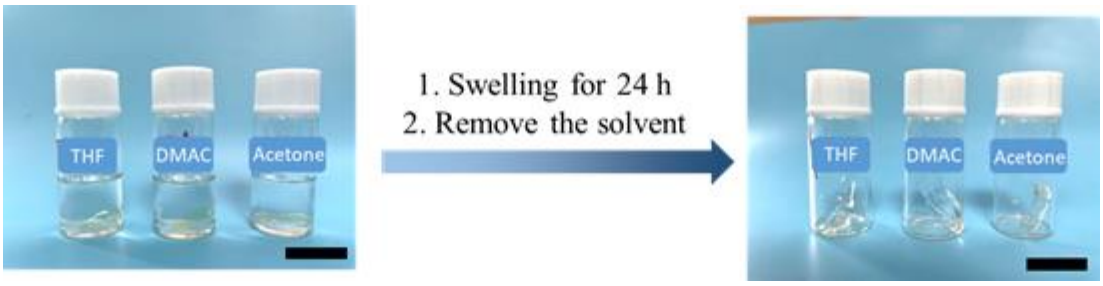

Figure S4. The I_{40}-SS-CPU was swollen in tetrahydrofuran (THF) dimethylacetamide (DMAC), and acetone (scale bar: 2 cm).

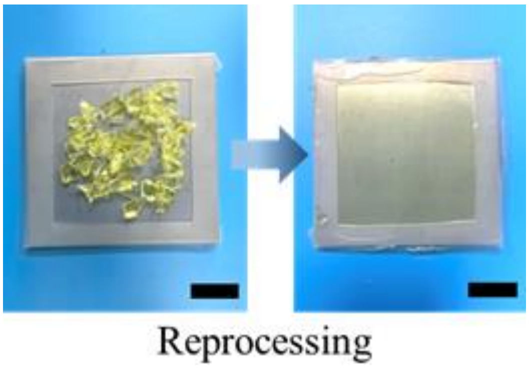

Figure S5. Photographs of reprocessing of the cut I_{40}-SS-CPU pieces by compression molding (Scale bar: 2 cm).

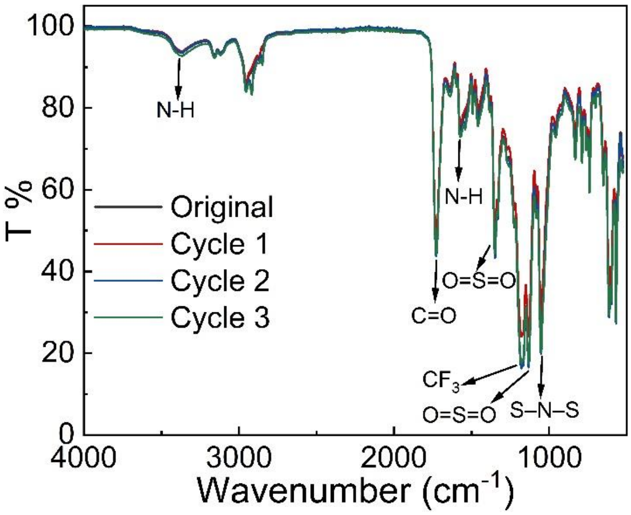

Figure S6. FTIR spectra of I_{40}-SS-CPU before and after reprocessing. The four vibrational bands located at 1346, 1180, 1133, and 1055 cm^{-1} corresponded to the O=S=O asymmetric, CF3, O=S=O symmetric, and S–N–S stretches of the [TFSI] anion in [EMI][TFSI], respectively. The vibrational bands of 3374, 1727, and 1503 cm^{-1} corresponded to N-H, C=O stretches, and N-H bending of polyurethane chain.

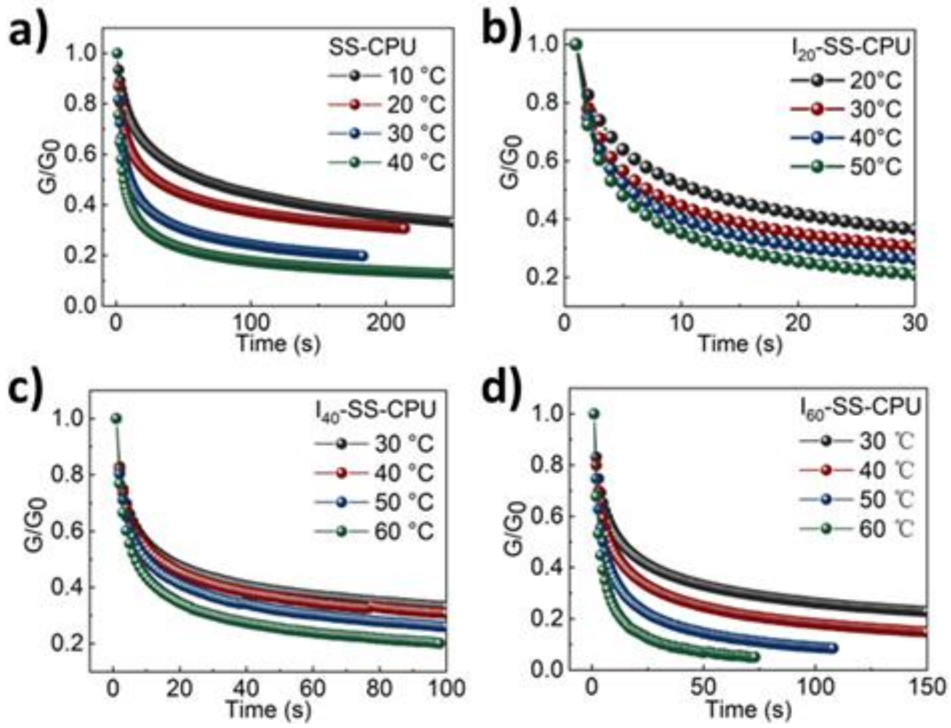

Figure S7 a–d) The stress-relaxation curves of a) SS-CPU, b) I_{20}-SS-CPU, c) I_{40}-SS-CPU, and d) I_{60}-SS-CPU.

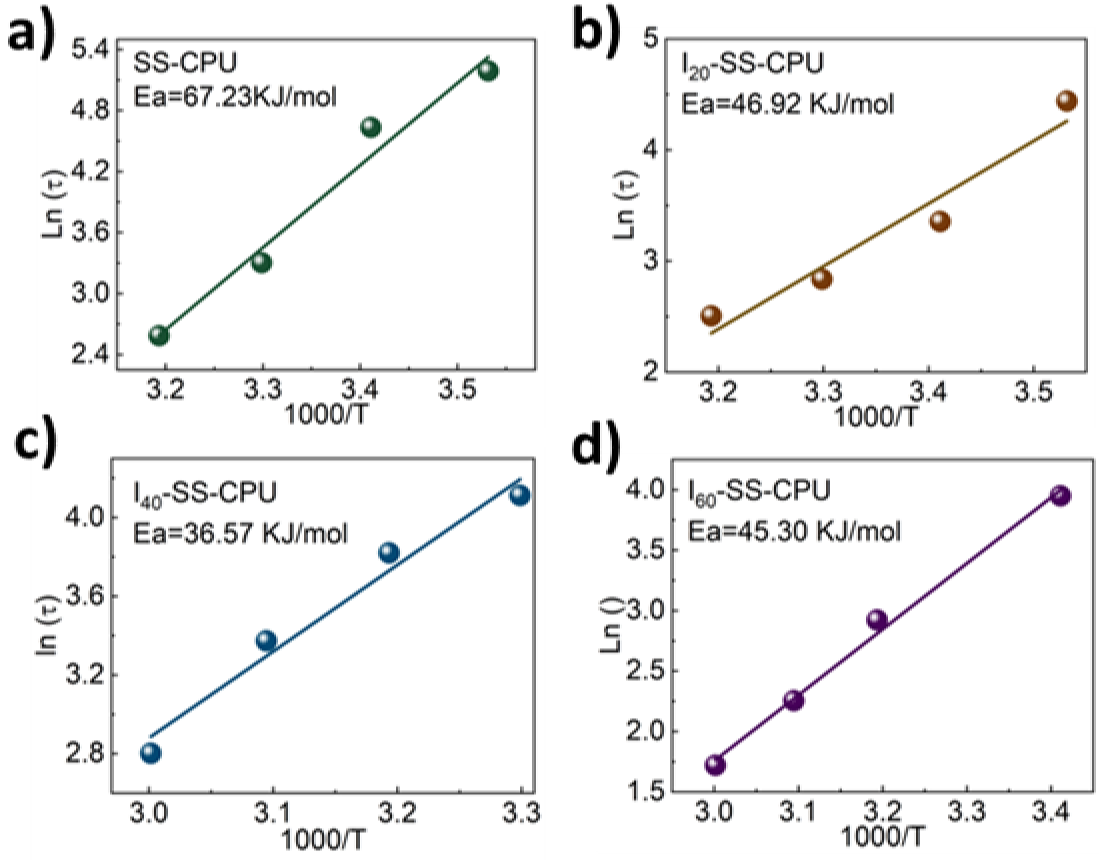

Figure S8. The relaxation times of a) SS-CPU, b) I_{20}-SS-CPU, c) I_{40}-SS-CPU, and d) I_{60}-SS-CPU fitted to the Arrhenius equation.

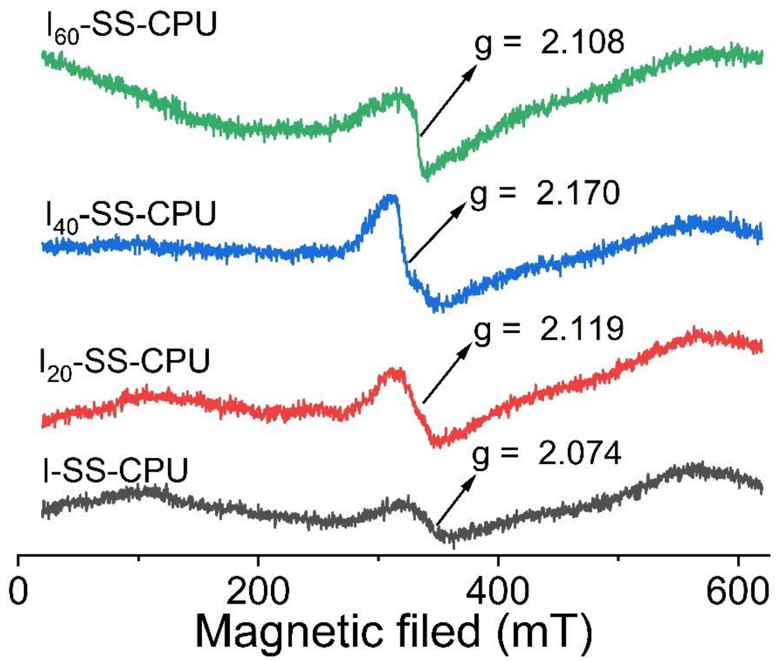

Figure S9. The first derivative of electron paramagnetic resonance spectra of SS-CPU and I-SS-CPUs.

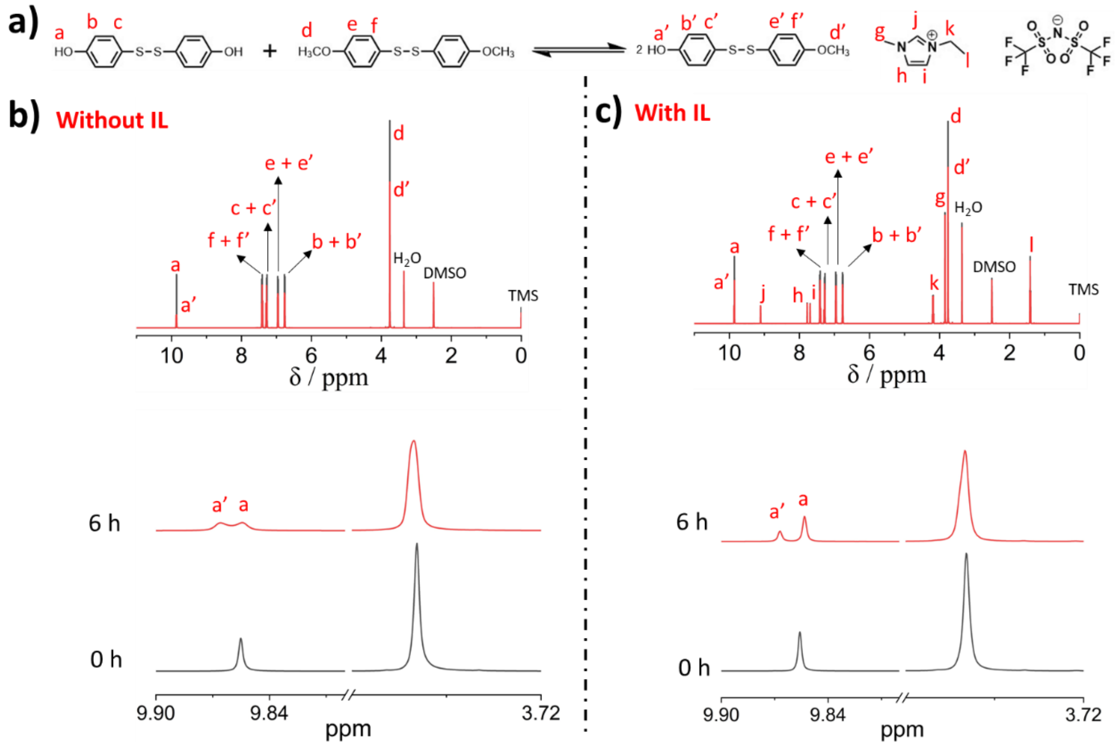

Figure S10. The dissociation kinetics of the disulfide bond with (left) and without (right) ionic liquid. (a) The molecular formula of small-molecule reaction model and ionic liquid. b) ^{1}H-NMR spectrum of the mixture of A (50.1 mg, 0.20 mmol), B (55.7 mg, 0.020 mmol), and DMSO-D_6 (0.6 ml) in 0 and 6 hours. The consumption rate of

A was used to calculate the dissociation rate of the disulfide bond with the following equation:

$$k_{-1} = -\frac{\ln\frac{[A]}{[A]_0}}{T} = -\frac{\ln 0.543}{6\ h} = 0.102\ h^{-1}$$

c) ^{1}H-NMR spectrum of the mixture of A (50.1 mg, 0.20 mmol), B (55.7 mg, 0.020 mmol), [EMI][TFSI] (100.0 mg, 0.26 mmol) and DMSO-D_6 (0.6 ml) in 0 hour and 6 hours. The consumption rate of A was used to calculate the dissociation rate of the disulfide bond with the following equation:

$$k_{-1} = -\frac{\ln\frac{[A]}{[A]_0}}{T} = -\frac{\ln 0.672}{6\ h} = 0.066\ h^{-1}$$

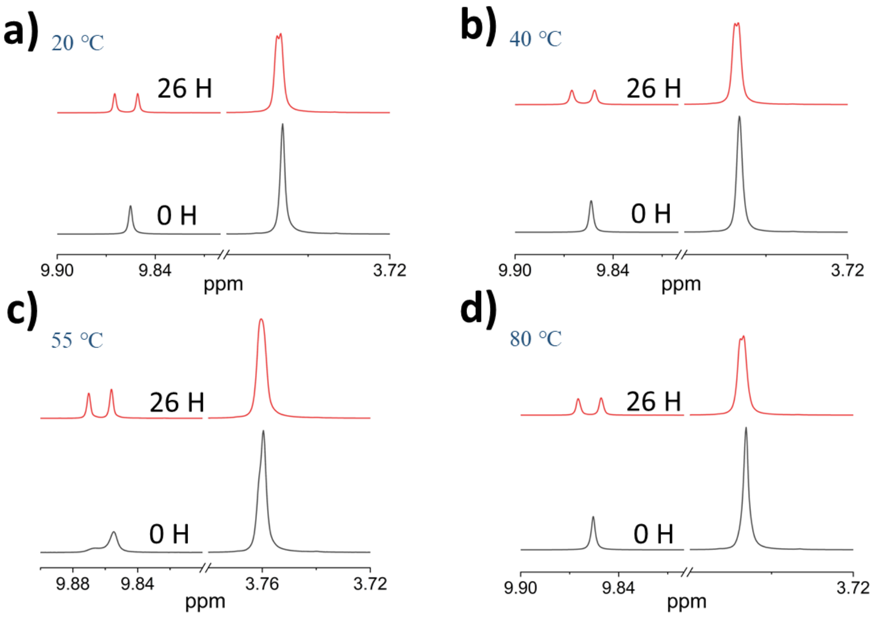

e)

T (°C)	$[A]_0$ (M)	$[B]_0$ (M)	$[A]_{eq}$ (M)	$[B]_{eq}$ (M)	$[C]_{eq}$ (M)	K_{eq}
20	0.0333	0.0328	0.0168	0.0164	0.0165	0.9881
40	0.0333	0.0330	0.0169	0.0166	0.0163	0.9471
55	0.0333	0.0284	0.0183	0.0133	0.0151	0.9368
80	0.0333	0.0316	0.0175	0.0158	0.0159	0.9143

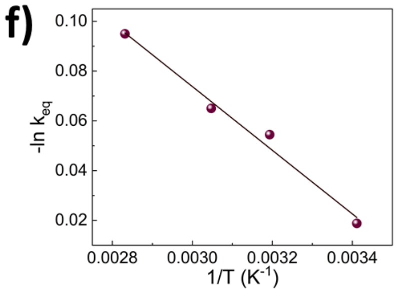

Figure S11. Thermodynamic equilibrium constants of the disulfide bond as a function

of temperatures. a-d) ^{1}H NMR spectra of the mixture of compound A and B at different temperatures in 0 h and 26 h. e) Concentrations of compounds A, B, and C with calculated equilibrium constants at different temperatures. f) Plot of linear regression of the logarithm of the equilibrium constant $\ln K_{eq}$ reciprocal of temperature 1/T. The equilibrium reaction follows van't Hoff equation with thermodynamic constant calculated as follows: $\Delta H = -1.06$ kJ mol^{-1}; $\Delta S = 3.80$ J mol^{-1} K^{-1}.

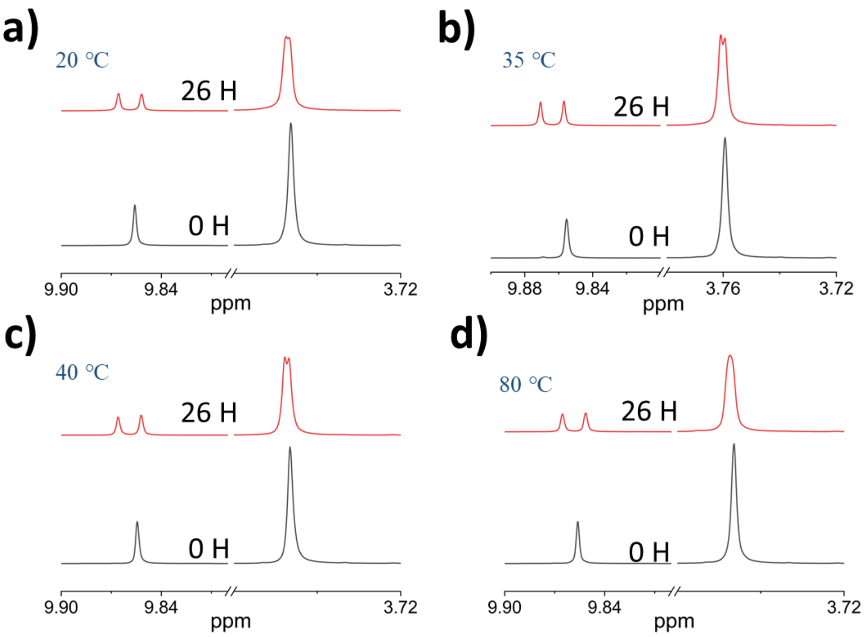

e)

T (°C)	$[A]_0$ (M)	$[B]_0$ (M)	$[A]_{eq}$ (M)	$[B]_{eq}$ (M)	$[C]_{eq}$ (M)	K_{eq}
20	0.0333	0.0342	0.0163	0.0172	0.0169	1.0187
35	0.0333	0.0330	0.0167	0.0167	0.0166	0.9881
40	0.0333	0.0294	0.0178	0.0139	0.0155	0.9710
80	0.0333	0.0327	0.0171	0.0156	0.0161	0.9189

f)

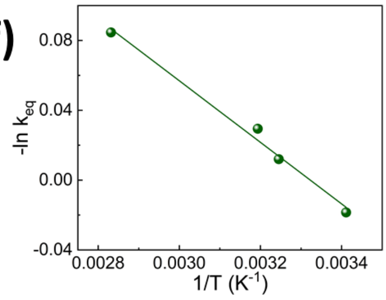

Figure S12. Thermodynamic equilibrium constants of the disulfide bond as a function

of temperatures. a-d) ^{1}H NMR spectra of the mixture of compound A, B, and [EMI][TFSI] at different temperatures in 0 h and 26 h. e) Concentrations of A, B, and C with calculated equilibrium constants at different temperatures. f) Plot of linear regression of the logarithm of the equilibrium constant $\ln K_{eq}$ reciprocal of temperature 1/T. The equilibrium reaction follows van't Hoff equation with thermodynamic constant calculated as follows: $\Delta H = -1.46$ kJ mol^{-1}; $\Delta S = 4.87$ J mol^{-1} K^{-1}.
